# Effects of Nicotine on the Central Nervous System and Sleep Quality in Relation to Other Stimulants: A Narrative Review

**DOI:** 10.7759/cureus.49162

**Published:** 2023-11-21

**Authors:** Nihaal Singh, Anil Wanjari, Arya Harshyt Sinha

**Affiliations:** 1 Medicine, Jawaharlal Nehru Medical College, Datta Meghe Institute of Higher Education and Research, Wardha, IND

**Keywords:** tobacco, addiction, sleep, central nervous system, nicotine

## Abstract

Nicotine is used extensively across the globe despite the common awareness of the fact that it might stimulate the neurological system in those who indulge in its consumption. Nicotine can be consumed in a wide number of various forms and can also be delivered in a wide variety of different ways. After it has been heated, it can be smoked, consumed sublingually, or brought into touch with mucosal surfaces, with the buccal mucosa being the most popular one. These three methods of consumption account for the vast majority of its use. It has been demonstrated without a reasonable doubt that people who partake in nicotine do, in fact, experience an increase in their levels of alertness, wakefulness, attention, and focus. The half-life of the substance, in addition to its effects, is highly variable depending on the forms in which it is consumed, viz. cigarettes, tobacco, gums, lozenges, and the manner in which it is administered. This is the case regardless of whether the chemical is administered orally or intravenously. It is common for a person to require multiple "hits" of the chemical throughout the course of the day, though the frequency of these needs and the intervals between them can vary greatly. The time interval between each of these "hits" can range anywhere from a few hours to a few minutes. The user has the potential to develop a tolerance to the neurostimulatory and systemic effects of nicotine, as well as a heightened sensitivity to those effects, and even hereditary predispositions to specific adverse consequences. There is also a possibility that the user will develop an addiction to nicotine. This literature review aims to explore the relationship between nicotine consumption and its effects on the central nervous system, especially on sleep.

## Introduction and background

Nicotine is a naturally occurring plant alkaloid typically found in the leaves of the tobacco plant. It is a neurologically active substance that notably stimulates the central nervous system (CNS) and the cardiovascular system [1,2]. Its effects can vary from person to person but generally include increased alertness, wakefulness, and, in some cases, enhanced creativity [3]. While there are regulations governing nicotine use and distribution, policies regarding its use are generally lenient. It is important to note that some nations have recently attempted to introduce stricter regulations regarding the sale of nicotine products to young and adolescent demographics [4,5].

Nicotine users often develop strong dependencies, both physically and psychologically, leading to addiction. Overcoming a nicotine habit is notoriously challenging, with some individuals remaining lifelong users well into old age [6]. There is a consensus in the scientific community that nicotine addiction is a significant issue due to its potential to lead to various health problems, including emphysema, coronary artery disease, and lung or oral mucosa carcinomas [7,8]. Prolonged nicotine use is well-documented to accelerate the formation of atherosclerotic plaques and induce hypercoagulable states, such as disseminated intravascular coagulation (DIC) [9]. Additionally, it is considered a major contributing factor to the development of cerebrovascular accidents in middle-aged users [10].

Numerous sleep-related disorders, such as insomnia, sleep apnea, nocturnal breathing issues, and poor sleep quality, have been linked to cigarette smoking [11]. The symptoms of these sleep disorders include difficulty falling asleep, trouble staying asleep, shorter sleep durations, and later sleep onset times [12,13]. Most notably, studies have shown that getting insufficient sleep increases the likelihood of developing long-term illnesses such as heart disease, diabetes, depression, and obesity [14].

While the precise nature of the relationship between smoking behavior and sleep quality is still largely unknown, understanding it is essential for developing treatment plans that will increase the likelihood of smokers successfully quitting [15,16]. It has been shown that a number of smoking-related factors, such as cravings, withdrawal symptoms, regular cigarette use, and nicotine dependency, are predictive of both successful quitting and relapse [17,18].

Remarkably, a recent study's unexpected conclusion implies that smokers who puff more might sleep better at night [19,20]. This unexpected result implies that heavier smokers might smoke more frequently, whereas lighter smokers might smoke at different times of the day [21]. People's capacity to sleep through the night may be significantly impacted by changes in the way nicotine is administered over time [22,23].

It has also been shown that exercise lessens the negative effects of smoking; however, research on this topic is still in its early stages [24]. Some studies have demonstrated that brief bursts of high-intensity aerobic exercise significantly reduce cravings for cigarettes, withdrawal symptoms, and the urge to smoke [25,26], though the precise mechanisms underlying the relationship between exercise and smoking behavior are still unknown [27]. One strategy that could be employed is stress reduction; exercise has the ability to indirectly improve smokers' sleep, which may then influence their smoking habits [28,29].

Nicotine, a main component of cigarettes, agonistically activates nicotinic cholinergic receptors (nAChRs) in the CNS and peripheral nervous system (PNS) [30,31]. A variety of α and β subunits make up these receptors; the most prevalent subtypes in the human brain are α3β4, which is heterogenic, α7 which is homomeric, and α4β2 [32]. Neurotransmitters such as norepinephrine, dopamine, γ-aminobutyric acid, glutamate, and serotonin are released when CNS is stimulated by nicotine or acetylcholine-induced nAChR, altering brain activity [33,34]. Thus, alterations in nAChR expression or function linked to disease may have an impact on neurotransmitter release, which, in turn, may have an impact on brain function [35,36].

Recognizing that nicotine is a naturally occurring plant chemical, primarily found in tobacco leaves, is also crucial. Because of its neuroactive properties, it stimulates CNS and the cardiovascular system. Nicotine generally promotes alertness and wakefulness, and occasionally sparks creative thought, though individual usage varies [37]. Despite recent attempts in several countries to enact more stringent laws aimed at limiting juvenile and adolescent populations' access to nicotine products, many places have relatively lax laws governing the use and distribution of nicotine [38].

There is a strong likelihood that consuming nicotine will lead to both physical and mental dependence, as well as addiction. Giving up the drug is a notoriously tough habit to break because some people use nicotine constantly all their lives, even into old age [39]. Science has generally agreed that nicotine addiction is a dangerous problem because it can lead to various health problems such as respiratory disorders (e.g., emphysema), different types of carcinomas, and cardiovascular disorders (e.g., coronary artery disease). Long-term nicotine use has been demonstrated to hasten the onset of atherosclerotic plaques and induce hypercoagulable illnesses such as DIC [40]. Furthermore, it is recognized that it plays a significant role in the prevalence of cerebrovascular accidents among middle-aged users.

## Review

Methodology

Literature Search

We used various databases such as PubMed, Cochrane Library, and Google Scholar. Our search included specific keywords relevant to our study, including “nicotine," "central nervous system," "sleep," "addiction," and "tobacco.” We also manually searched the reference lists of relevant articles to identify additional studies.

Inclusion and Exclusion Criteria

We incorporated in our research studies that explored the various aspects of nicotine, such as tobacco, addiction, sleep, and CNS. However, we opted to exclude non-English studies and those that did not undergo the peer-review process.

Data Extraction and Synthesis

In order to conduct our analysis, we obtained information from each study that was included in the research. To evaluate this collected information effectively, we employed a narrative approach, which allowed us to provide an overview while highlighting important and current details.

Data Analysis

**Figure 1:**
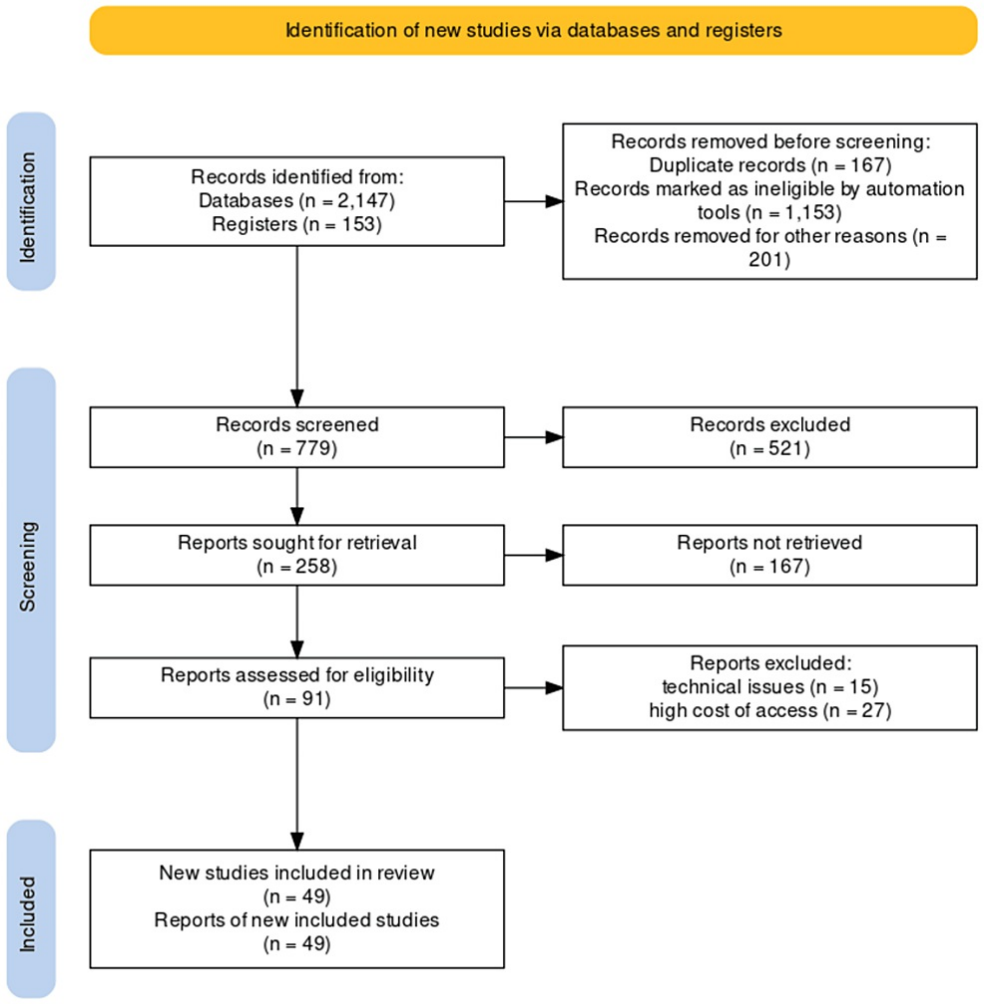
Search methodology flow diagram [41]

In our analysis, we employed a qualitative method to examine the data. We focused on identifying recurring themes and patterns that emerged from the studies included in our research. Furthermore, we used descriptive statistics as a means of succinctly summarizing the findings obtained. Figure 1 shows the flow diagram of the literature search.

The effect of nicotine consumption on the central nervous system

3-(1-Methylpyrrolidin-2-yl) pyridine is the scientific name for nicotine, a well-known alkaloid, which is mainly found in tobacco leaves [1,2]. Its chronic consumption is strongly associated with several harmful health consequences, including the development of heart and lung conditions, an increased risk of cancer, and an increased susceptibility to infectious diseases such as TB, chlamydia, and other STDs [4]. Recent research, however, suggests that nicotine may also be advantageous to health, particularly in terms of cognitive function [30].

Nicotine agonists initially bind to PNS and CNS through nAChRs [32,35,36]. These nAChRs, which are made up of various combinations of five α and β subunits, are thought to contain 9 α-subunits and 3 β-subunits, which account for the diversity of receptor subtypes. The human brain is known to contain the receptor subtypes α4β2, α3β4 (heterogenic), and α7 (homomeric). More specifically, homomeric α7 nAChR is linked to learning, synaptic transmission, and sensory gating, whereas α3β4 nAChRs mediate the cardiovascular system's effects of nicotine. Numerous neurotransmitters, including glutamate, dopamine, serotonin, norepinephrine, and γ-aminobutyric acid, are released under the control of CNS nAChRs when they are stimulated by nicotine or acetylcholine [34]. Changes in nAChR expression or function, often associated with illnesses, may throw off the delicate neurotransmitter-release balance. This could have an effect on how the brain works.

Effect of Chronic Nicotine Abuse on Neurotransmitters

Long-term exposure to nicotine is known to desensitize people to nAChR, which in otherwise healthy individuals impairs cognitive function. The phosphodiesterase-5 (PDE-5) signaling mechanism and the downregulation of estrogen production are just two of the many underlying mechanisms that may be in charge of this nicotine-induced cognitive impairment. Nicotine primarily causes the cleavage of two vital molecules, cyclic guanosine monophosphate and cyclic adenosine monophosphate (PDE-5), which are involved in downstream signaling pathways that lead to memory impairment. Furthermore, the enzyme aromatase, also referred to as estrogen synthase, which is necessary for the brain's synthesis of estrogen, is blocked by nicotine. Estrogen stimulates a wide range of neurotransmitters, such as noradrenaline, acetylcholine, serotonin, and glutamate. As a result, the estrogen receptors in the brain become more active, increasing the hormone's levels. Significantly activating the brain circuits required for memory encoding are these neurotransmitters [25]. Consequently, exposure to nicotine modifies the biosynthesis of estrogen, which affects cognitive function in healthy individuals when combined with nicotine's effects on elevated PDE-5 levels.

**Figure 2:**
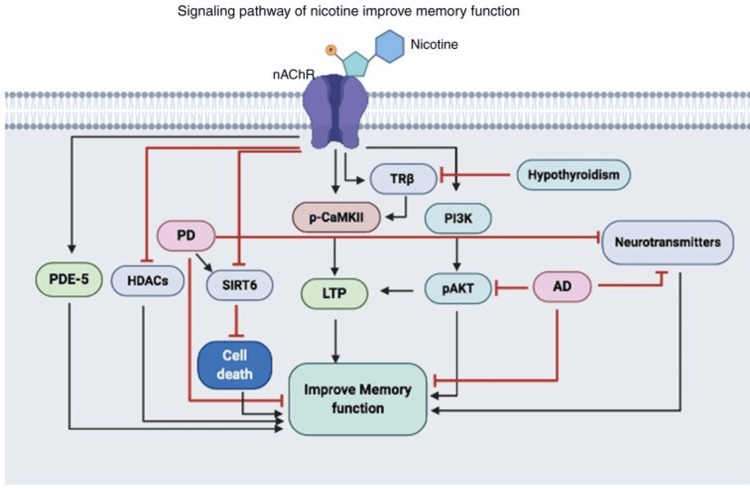
Signaling pathway of nicotine improves memory function PDE-5, phosphodiesterase-5; HDAC, histone deacetylases; PD, Parkinson's disease; SIRT6, Sirtuin 6; LTP, long-term potentiation; p-CAMKII, phosphorylated calmodulin-dependent protein kinase II; TRβ, thyroid receptor subunit β; PI3K, phosphoinositide 3-kinase; AD, Alzheimer's disease; nAChR, nicotinic cholinergic receptors [5] Note: Reproduced with appropriate permissions from Open Access journals providing unrestricted use with citations.

Nevertheless, despite nicotine's detrimental effects on cognitive function, some scientific research indicates that it may enhance memory and learning processes. Figure 2 shows the signaling pathway of nicotine and its effects on memory function.

How nicotine affects sleeping patterns under normal conditions

Smokers often experience shorter and lower-quality sleep compared to non-smokers. These findings are consistent with prior research involving different demographics, which consistently shows that smokers tend to have poorer sleep quality than non-smokers [2].

Moreover, smoking is linked to various factors that influence the quality of sleep, encompassing subjective sleep quality, sleep efficiency, duration, and onset latency, as demonstrated by our research. This highlights that smoking exerts a diverse range of effects on sleep, not all attributable to a single factor. Nicotine, the primary component in cigarettes, serves as a significant contributor. Firstly, a physiological need for nicotine during the night may initially induce insomnia, preventing restful sleep. Secondly, consuming nicotine close to bedtime can lead to heightened alertness and delayed sleep onset due to its stimulant properties. Additionally, nicotine has been associated with issues such as snoring, obstructive sleep apnea, and disrupted circadian rhythms [12,19].

Across all age groups, a higher percentage of smokers reported difficulty falling asleep, which may have long-term implications. Similar trends were observed in a study involving Thai college students: smokers reported longer sleep durations, increased use of sleep aids, and daytime dysfunction due to insufficient sleep. Furthermore, smokers consistently reported lower sleep quality, regardless of differences in their place of residence, marital status, or level of education [21].

A recent study uncovered an intriguing connection between higher cigarette consumption among smokers and improved sleep quality. Interestingly, heavier smokers did not exhibit a similar link with insomnia. However, this finding aligns with a surprising discovery made by researchers: individuals who consumed fewer than 15 cigarettes daily tended to have shorter overall sleep durations. This could be attributed to the fact that light smokers tend to use tobacco sporadically throughout the day, whereas chronic smokers adhere to a more consistent smoking schedule. This implies that the timing of nicotine intake may influence one's nighttime sleep quality [23,42].

Nevertheless, a positive correlation emerged between the lower sleep quality experienced by smokers and factors such as the duration of their smoking history, the frequency of their quit attempts, and the intensity of their cravings. Prolonged smoking can impact physiological systems that evolve with ongoing nicotine exposure over time. Research on animals has shown that both acute and chronic exposure to cigarette smoke can disrupt clock genes and natural circadian rhythms [35]. Smokers who are making a concerted effort to quit may not be satisfied with the consequences of smoking, including poor sleep quality. This could indicate that they require assistance in quitting to attain better sleep. Poor sleep quality is strongly associated with self-efficacy in quitting addictive substances and often correlates with a higher likelihood of relapse in individuals attempting to quit [36,37]. Given these findings, sleep therapy has been suggested as a complementary approach to other forms of therapy to aid smokers in their cessation efforts. Cravings for cigarettes can underscore individual differences in the body's response to nicotine and underscore the significance of sleep disturbances in the withdrawal process, especially for those experiencing "nocturnal sleep-disturbing nicotine craving," characterized by nighttime cigarette cravings [38].

A recent study on young adult smokers revealed similar correlations between cigarette smoking, withdrawal symptoms, cravings, and sleep quality [14]. However, the data yielded conflicting conclusions in some respects. Specifically, the findings contradicted the statistically significant relationship between cravings and sleep quality, as observed in well-known sleep studies. All considered studies did agree that individuals facing sleep difficulties tended to experience more withdrawal symptoms.

Furthermore, the results in the above study diverged from the more popular sleep research, which indicated that smokers with higher nicotine dependence and heavier smoking habits were more likely to have lower sleep quality. Surprisingly, the data did not establish a statistically significant connection between daily cigarette consumption and the quality of sleep. Several plausible explanations exist for the relationship between the adverse effects of smoking and inadequate sleep. These include the impact of nicotine on circadian rhythms, nighttime cravings, withdrawal symptoms, and exposure to secondhand smoke. Another reasonable explanation for the link between withdrawal symptoms and sleep quality is that the Minnesota Nicotine Withdrawal Scale (MNWS) encompasses various sections related to sleep, such as sleep disturbances and insomnia. These aspects may account for the observed association between withdrawal and poor sleep quality [43].

Researchers conducted a detailed comparison between our sleep quality measurements and the MNWS items, item by item. The results indicated that five out of the seven withdrawal subscales, even those not directly related to sleep, exhibited a statistically significant correlation with overall sleep quality. This suggests that a connection between withdrawal symptoms and sleep quality might extend beyond the MNWS's sleep-related items.

Analyzing nicotine with respect to other stimulants as variables that impact healthy sleep

**Figure 3:**
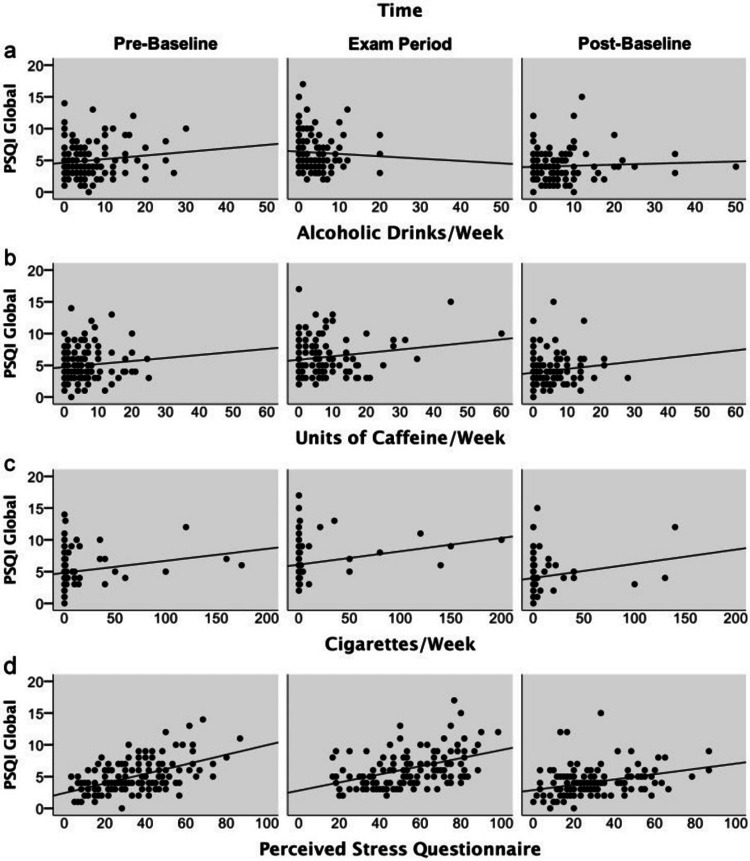
Amounts of alcohol (a), caffeine (b), nicotine (c), as well as perceived stress (d) plotted against PSQI global scores within a week. PSQI, Pittsburgh Sleep Quality Index [19] Note: Reproduced with appropriate permissions from Open Access journals providing unrestricted use with citations.

By examining factors such as duration, gender, age, fitness, alcohol and caffeine consumption, nicotine use, and perceived stress, we identified the factors influencing sleep quality (Figures 3a, 3b, 3d). The most significant predictor of overall sleep quality was found to be perceived stress, which had a moderate but significant impact [19].

Higher levels of perceived stress were associated with lower sleep quality. Additionally, there was evidence of a connection between increased cigarette consumption per week and poorer sleep quality, although this impact was relatively minor. However, no clear relationship was observed between variations in the Pittsburgh Sleep Quality Index (PSQI) global score and attributes such as age, gender, current health status, and alcohol and caffeine intake [44,45].

Importantly, when comparing the PSQI global score during the pre-baseline and exam period to the post-baseline period, this difference persisted even after adjusting for age, gender, health status, use of legal drugs, and perceived stress. The effects of time and perceived stress had a moderate impact, explaining 13.9% of the total variation. For example, perceived stress alone accounted for 8.55% of the unique variance, whereas time explained 2.41% of the variance [19].

Figure 3 illustrates how perceived stress levels (Figure 3d) over the previous week correlate with the PSQI global scores. The amounts of alcohol (Figure 3a), caffeine (Figure 3b), and nicotine (Figure 3c) consumed are also plotted against the PSQI global scores. Notably, the major predictors of the PSQI global score were the level of nicotine use and perceived stress levels, indicating a significant influence of these variables on sleep quality. However, the overall PSQI scores did not display any discernible relationship with alcohol or caffeine consumption [46].

Studies have shown that perceived stress has a substantial impact on sleep quality, with higher stress levels correlating with poorer sleep. Weekly cigarette smoking also negatively affects sleep quality. Importantly, sleep quality seems unrelated to age, gender, health status, or coffee and alcohol consumption. Significant correlations exist between perceived stress, nicotine use, and sleep quality, whereas no connection is evident between alcohol or caffeine usage and PSQI.

In Figure 3, the results of the mixed model are presented along with various model parameters and their respective units. A higher PSQI score is associated with positive beta values, indicating a decline in sleep quality [47]. The mean served as the center for all the covariates [48]. By focusing on the two main predictors of sleep quality - perceived stress and nicotine use - we gain more insight into the variables that impact sleep quality. These data offer valuable information about the complex interactions between these variables and how they affect sleep, helping us better understand the underlying relationships [49,50]. Figure 3 shows amounts of alcohol (Figure 3a), caffeine (Figure 3b), nicotine (Figure 3c), and perceived stress (Figure 3d) plotted against PSQI global scores within a week.

## Conclusions

Tobacco leaves contain nicotine, a stimulant affecting the heart and CNS. Some claim that it boosts alertness, vigilance, and creativity. Due to its potential impact on productivity, nicotine's use and distribution are regulated, with recent efforts focusing on tighter controls, especially for minors. Nicotine can lead to strong physical and psychological addiction, which is often lifelong. Medical consensus confirms that nicotine addiction can result in conditions such as emphysema, coronary artery disease, and lung or oral carcinomas. Prolonged nicotine use accelerates the development of health issues such as hypercoagulable disorders, atherosclerotic plaques, and cerebrovascular accidents, particularly in middle-aged users. Smoking significantly influences sleep patterns, affecting latency, duration, quality, and habitual efficiency. Nicotine, the primary cigarette component, disrupts sleep by causing disturbances when used before bedtime and due to nighttime cravings. It also leads to irregular circadian rhythms, snoring, and obstructive sleep apnea. Sleep difficulties, such as difficulty falling asleep, are common among smokers of all ages, with potential long-term consequences. Regardless of location, marital status, or education level, smokers generally experience lower-quality sleep.

Recent research involving young adult smokers has identified links between smoking, cravings, withdrawal, and sleep. While some findings align with earlier studies, others do not. Notably, withdrawal symptoms are more common among those who experience insufficient sleep, but regular cigarette smoking does not seem to appreciably impact sleep quality. In summary, smoking's negative impact on sleep quality is attributed to nicotine's effects on circadian rhythms, nighttime cravings, withdrawal symptoms, and secondhand smoke exposure.

## References

[1] Allen AM, Abdelwahab NM, Carlson S, Bosch TA, Eberly LE, Okuyemi K (2018). Effect of brief exercise on urges to smoke in men and women smokers. Addict Behav.

[2] Allen A, Carlson SC, Bosch TA, Eberly LE, Okuyemi K, Nair U, Gordon JS (2018). High-intensity interval training and continuous aerobic exercise interventions to promote self-initiated quit attempts in young adults who smoke: feasibility, acceptability, and lessons learned from a randomized pilot trial. J Addict Med.

[3] Bastien CH, Vallières A, Morin CM (2001). Validation of the insomnia severity index as an outcome measure for insomnia research. Sleep Med.

[4] Bittoun R (2008). Carbon monoxide meter: the essential clinical tool — the “stethoscope” — of smoking cessation. J Smok Cessat.

[5] Alhowail A (2021). Molecular insights into the benefits of nicotine on memory and cognition (Review). Mol Med Rep.

[6] AlRyalat SA, Kussad S, El Khatib O, Hamad I, Al-Tanjy A, Alshnneikat M, AbuMahfouz B (2021). Assessing the effect of nicotine dose in cigarette smoking on sleep quality. Sleep Breath.

[7] Caviness CM, Anderson BJ, Stein MD (2019). Impact of nicotine and other stimulants on sleep in young adults. J Addict Med.

[8] Garcia AN, Salloum IM (2015). Polysomnographic sleep disturbances in nicotine, caffeine, alcohol, cocaine, opioid, and cannabis use: a focused review. Am J Addict.

[9] Hamberger ES, Halpern-Felsher B (2020). Vaping in adolescents: epidemiology and respiratory harm. Curr Opin Pediatr.

[10] Irish LA, Kline CE, Gunn HE, Buysse DJ, Hall MH (2015). The role of sleep hygiene in promoting public health: a review of empirical evidence. Sleep Med Rev.

[11] Jaehne A, Loessl B, Bárkai Z, Riemann D, Hornyak M (2009). Effects of nicotine on sleep during consumption, withdrawal and replacement therapy. Sleep Med Rev.

[12] Jarvik ME (1991). Beneficial effects of nicotine. Br J Addict.

[13] Jähne A, Cohrs S, Rodenbeck A (2010). [Nicotine. Influence on sleep and its relevance for psychiatry and psychotherapy]. Nervenarzt.

[14] Leonel LF, Morelhão PK, Tufik S, Andersen ML (2020). Sleep disturbances and nicotine addiction:a bidirectional relationship?. Sleep Breath.

[15] O'Reilly C, Chapotot F, Pittau F, Mella N, Picard F (2019). Nicotine increases sleep spindle activity. J Sleep Res.

[16] Purani H, Friedrichsen S, Allen AM (2019). Sleep quality in cigarette smokers: associations with smoking-related outcomes and exercise. Addict Behav.

[17] Soreca I, Conklin CA, Vella EJ (2022). Can exercise alleviate sleep disturbances during acute nicotine withdrawal in cigarette smokers?. Exp Clin Psychopharmacol.

[18] Vieyra Reyes P (2009). Acción de la Nicotina Como antidepresivo y regulador del sueño en sujetos deprimidos. Rev Neurol.

[19] Zunhammer M, Eichhammer P, Busch V (2014). Sleep quality during exam stress: the role of alcohol, caffeine and nicotine. PLoS One.

[20] Branstetter SA, Horton WJ, Mercincavage M, Buxton OM (2016). Severity of nicotine addiction and disruptions in sleep mediated by early awakenings. Nicotine Tob Res.

[21] Salin-Pascual RJ (2006). Effects of nicotine replacement therapies on sleep. Sleep Med.

[22] Sharma R, Lodhi S, Sahota P, Thakkar MM (2015). Nicotine administration in the wake-promoting basal forebrain attenuates sleep-promoting effects of alcohol. J Neurochem.

[23] Buysse DJ, Reynolds CF, Monk TH (1989). The Pittsburgh Sleep Quality Index: a new instrument for psychiatric practice and research. Psychiatry.

[24] Chennaoui M, Arnal PJ, Sauvet F, Léger D (2015). Sleep and exercise: a reciprocal issue?. Sleep Med Rev.

[25] Cohrs S, Rodenbeck A, Riemann D (2014). Impaired sleep quality and sleep duration in smokers-results from the German Multicenter Study on Nicotine Dependence. Addict Biol.

[26] Cox LS, Tiffany ST, Christen AG (2001). Evaluation of the brief questionnaire of smoking urges (QSU-brief) in laboratory and clinical settings. Nicotine Tob Res.

[27] Daniel JZ, Cropley M, Fife-Schaw C (2006). The effect of exercise in reducing desire to smoke and cigarette withdrawal symptoms is not caused by distraction. Addiction.

[28] Daniel J, Cropley M, Ussher M, West R (2004). Acute effects of a short bout of moderate versus light intensity exercise versus inactivity on tobacco withdrawal symptoms in sedentary smokers. Psychopharmacology (Berl).

[29] Benowitz NL, Hukkanen J, Jacob P 3rd (2009). Nicotine chemistry, metabolism, kinetics and biomarkers. Handb Exp Pharmacol.

[30] Broide RS, Winzer-Serhan UH, Chen Y, Leslie FM (2019). Distribution of α7 nicotinic acetylcholine receptor subunit mRNA in the developing mouse. Front Neuroanat.

[31] Mishra A, Chaturvedi P, Datta S, Sinukumar S, Joshi P, Garg A (2015). Harmful effects of nicotine. Indian J Med Paediatr Oncol.

[32] Bagaitkar J, Demuth DR, Scott DA (2008). Tobacco use increases susceptibility to bacterial infection. Tob Induc Dis.

[33] Unwin N (2013). Nicotinic acetylcholine receptor and the structural basis of neuromuscular transmission: insights from Torpedo postsynaptic membranes. Q Rev Biophys.

[34] Gotti C, Zoli M, Clementi F (2006). Brain nicotinic acetylcholine receptors: native subtypes and their relevance. Trends Pharmacol Sci.

[35] Dani JA (2015). Neuronal nicotinic acetylcholine receptor structure and function and response to nicotine. Int Rev Neurobiol.

[36] Hone AJ, McIntosh JM (2018). Nicotinic acetylcholine receptors in neuropathic and inflammatory pain. FEBS Lett.

[37] Zaveri N, Jiang F, Olsen C, Polgar W, Toll L (2010). Novel α3β4 nicotinic acetylcholine receptor-selective ligands. Discovery, structure-activity studies, and pharmacological evaluation. J Med Chem.

[38] Aberger K, Chitravanshi VC, Sapru HN (2001). Cardiovascular responses to microinjections of nicotine into the caudal ventrolateral medulla of the rat. Brain Res.

[39] Levin ED, Bettegowda C, Blosser J, Gordon J (1999). AR-R17779, and alpha7 nicotinic agonist, improves learning and memory in rats. Behav Pharmacol.

[40] Peixoto CA, Nunes AK, Garcia-Osta A (2015). Phosphodiesterase-5 inhibitors: action on the signaling pathways of neuroinflammation, neurodegeneration, and cognition. Mediators Inflamm.

[41] Haddaway NR, Page MJ, Pritchard CC, McGuinness LA (2022). PRISMA2020: An R package and Shiny app for producing PRISMA 2020-compliant flow diagrams, with interactivity for optimised digital transparency and Open Synthesis. Campbell Syst Rev.

[42] Lund HG, Reider BD, Whiting AB, Prichard JR (2010). Sleep patterns and predictors of disturbed sleep in a large population of college students. J Adolesc Health.

[43] Zunhammer M, Eberle H, Eichhammer P, Busch V (2013). Somatic symptoms evoked by exam stress in university students: the role of alexithymia, neuroticism, anxiety and depression. PLoS One.

[44] Ahrberg K, Dresler M, Niedermaier S, Steiger A, Genzel L (2012). The interaction between sleep quality and academic performance. J Psychiatr Res.

[45] Astill RG, Verhoeven D, Vijzelaar RL, Van Someren EJ (2013). Chronic stress undermines the compensatory sleep efficiency increase in response to sleep restriction in adolescents. J Sleep Res.

[46] Steptoe A, Wardle J, Pollard TM (1996). Stress, social support and health-related behavior: a study of smoking, alcohol consumption and physical exercise. J Psychosom Res.

[47] Oaten M, Cheng K (2006). Improved self-control: The benefits of a regular program of academic study. Basic Appl Soc Psych.

[48] Oaten M, Cheng K (2005). Academic examination stress impairs self-control. J Soc Clin Psychol.

[49] West R, Lennox S (1992). Function of cigarette smoking in relation to examinations. Psychopharmacology (Berl).

[50] Drake C, Roehrs T, Shambroom J, Roth T (2013). Caffeine effects on sleep taken 0, 3, or 6 hours before going to bed. J Clin Sleep Med.

